# Editorial: Antimicrobial use, antimicrobial resistance, and the microbiome in animals, volume II

**DOI:** 10.3389/fvets.2023.1149010

**Published:** 2023-02-21

**Authors:** Moussa S. Darria, Xin Zhao, Patrick Butaye

**Affiliations:** ^1^Guelph Research and Development Center, Agriculture and Agri-Food Canada, Ottawa, ON, Canada; ^2^Department of Animal Science, McGill University, Montreal, QC, Canada; ^3^Department of Pathobiology, Pharmacology and Zoological Medicine Faculty of Veterinary Medicine, Ghent University, Merelbeke, Belgium

**Keywords:** antimycobacterial, resistance, microbiota, animals, alternatives to antibiotic

## Introduction

As demonstrated by the COVID-19 pandemic, endemic diseases or epidemic outbreaks represent a significant financial risk to society. In veterinary medicine, risks also include the loss of animals and the reduction of productivity and market access. Antimicrobials contribute to the treatment and prevention of infectious diseases in both animals and humans while improving farm animals' productivity and welfare. However, antimicrobial resistance (AMR) is becoming an important and growing economic and social problem associated with annual costs estimated at US$1 trillion to US$3.4 trillion worldwide (1, 2), and is regarded as the silent pandemic. Even though AMR, a global threat to humans, animals, and the environment, is complex, the wide use of antibiotics has been linked to the emergence and spread of AMR in all ecosystems (One Health). This Research Topic presents 12 studies on antimicrobial use (AMU) and AMR, as well as on antimicrobial impacts on the microbiota and epidemiology, dissemination-transmission, and surveillance of AMR.

## AMU and AMR

It is well known that antimicrobial resistant bacteria are selected mainly by antibiotics/antimicrobials use (AMU). In conventional production, antibiotics (although “antibiotics” and “antimicrobials” are sometimes used interchangeably, antibiotics are actually a subset of antimicrobials) have been used to prevent infectious diseases. However, this practice has been discontinued in more and more countries due to restrictions on antibiotic use. Antibiotics as feed additives to promote growth and eventually prevent diseases in healthy production animals were banned by the European Union in 2006. In 2018, the European Parliament approved new restrictions on the use of antimicrobials in healthy livestock. The Government of Canada (and also many other countries) has developed a Federal Framework and Federal Action Plan on AMR to initiate and take action in the areas of surveillance, stewardship, and innovation https://www.canada.ca/en/health-canada/services/publications/drugs-health-products/tackling-antimicrobial-resistance-use-pan-canadian-framework-action.html. This responsible use in animal production is intended to preserve the effectiveness of antibiotics and minimize the development and spread of AMR.

In this Research Topic, Lagarde et al. evaluated the impact of regulation by comparing the AMR situation in dairy cattle in Québec before and after the introduction of the new regulation. They found that AMR was significantly decreased in generic *Escherichia coli* from dairy cattle, and this started 2 years after the initiation of the AMU regulation. Despite this positive effect on AMR, decreasing AMU should also be evaluated for other aspects, including health and productivity. Another study on the reduction of antimicrobial use by Khine et al. showed a decline in pathogenic *mcr*-positive *E. coli* following the withdrawal of colistin in pigs. The study by Furuya et al. reported significantly higher resistance rates in *E. coli* and *Enterococcus* spp. isolates from sick pet animals than in those from healthy ones and concluded that the use of antimicrobials could select resistant *E. coli* and *Enterococcus* spp. The optimization of therapeutic doses by better understanding of pharmacokinetics and pharmacodynamics (PKs and PDs) could reduce the burden of the therapeutic use of antimicrobials on AMR. This concept has been presented for danofloxacin in pigs by Zhou et al..

### Microbiota and alternatives

The microbiota play critical roles in the gut and establish general health in the animal by maintaining/improving organ integrity and function and the provision and absorption of nutrients, as well as protecting against pathogens, including promoting immunity. A well-established microbiota plays a particularly important role in young animals. Feed additives, including alternatives to antibiotics, received attentions after the ban or restriction of in-feed antibiotics as growth promoters. Few studies have investigated the effects of antimicrobials on the metabolism, physiology, and immunity of animals. By contrast, several studies have reported their effects on microbiota and microbiomes. However, many other factors, such as genetics (line), physiological status, sex (male or female), health (clinical and subclinical), and housing/husbandry, influence the microbiota. Microbes respond to antimicrobials by developing and acquiring resistance mechanisms and changing gene expression patterns, which alter their metabolism and nutrient uptake and transport (4). The elimination or growth inhibition of bacteria by antibiotics results in changes in bacterial community structure and diversity.

In this Research Topic, the analysis of fecal microbiota in healthy, diarrheal, and treated weaned piglets showed differences between these three animal groups (Kong et al.). A comparison of the fecal microbiomes and antibiotic resistance genes (ARGs) in free-ranging and zoo-captive rhesus macaques by Jia et al., revealed that semi-captive wildlife might harbor a higher diversity of ARGs. Anemoside from *Pulsatillae Radix* has been found to potentially alleviate calf diarrhea, protect the integrity of the intestinal mucosa, and change the structure of intestinal microbiota (Lu et al.). Baicalin, another plant compound, this time from *Scutellaria baicalensis (*Baikal skullcap or Chinese skullcap), has been shown to potentially reverse azithromycin resistance in *Staphylococcus saprophyticus* (Wang et al.).

### Transmission, epidemiology, and surveillance

It is important to intensify research to understand the circumstances leading to the emergence of pathogenic bacteria and antibiotic resistance. In animal production industries, better practices with respect to food and environmental safety, as well as public and animal health and welfare, still need to be developed. Microorganisms living in changing environmental conditions adapt and evolve. Bacterial resistance determinants can be spread through horizontal gene transfer (HGT), which could be related to temperature (3). Therefore, climate change and AMR are interlinked and both should be addressed to protect humans, animals, and the environment.

“One Health” approaches, using “omics” and well-structured government-controlled surveillance, are needed. Additionally, it is imperative to understand the impacts of environmental factors on the evolution of bacteria and the development of AMR. In this context, Günther et al. investigated environmental factors associated with the prevalence of extended-spectrum beta-lactamase (ESBL) and AmpC-producing *E. coli* in wild boar. The findings from this study improved our understanding of the distribution of AMR in humans and animals using “One Health” approaches. The plasmid pE165, a mobile genetic element (MGE) involved in the horizontal transfer of *erm(T)* in *Enterococcus faecalis*, and its intraspecies and interspecies transmission ability, was determined by Li X-Y et al.. Different types of multidrug-resistant *Klebsiella pneumoniae* harboring virulence and resistance genes on MGEs, including IncFIB-, IncFII-, IncR-, and IncX3-type plasmids, were shown to be present in diseased dogs and cats in China (Zhang et al.). Furthermore, Li A et al. showed the simultaneous occurrence of *tet*(*X*4), *bla*_NDM − 1_, and *bla*_OXA − 58_ in a porcine *Acinetobacter towneri* isolate. The *tet(X4)* and the florfenicol *floR* resistance genes were flanked by IS91-like elements, emphasizing the importance of AMR surveillance in animals.

### Perspectives

Antimicrobial resistance is a “One Health” issue because AMR genes can be spread across humans, animals, and the environment. Surveillance, whole-genome sequencing, microbiota/microbiome, and antibiotic stewardship research are needed to determine important AMR drivers. The identification of hot spots and the ability to predict phenotype and transmission pathways, along with the adoption of best AMU practices, will help mitigate AMR. New knowledge contributing to the improvement of animal health and production, as well as studies providing science-based evidence about AMR transmission through the food chain and the environment, are needed. Owing to the high load of ARGs in animal manure and their potential spread to the environment when manures are used as soil fertilizers, the effects of different treatments of raw manures, such as composting (thermophilic composting and vermicomposting) and anaerobic digestion, should be investigated.

## Author contributions

All authors listed have made a substantial, direct, and intellectual contribution to the work and approved it for publication.
